# Effect on background checks of newly-enacted comprehensive background check policies in Oregon and Washington: a synthetic control approach

**DOI:** 10.1186/s40621-019-0225-8

**Published:** 2019-11-27

**Authors:** Alvaro Castillo-Carniglia, Daniel W. Webster, Garen J. Wintemute

**Affiliations:** 1 Society and Health Research Center, Universidad Mayor, Badajoz 130, office 1306, Las Condes, Santiago, Chile; 2 School of Public Health, Universidad Mayor, Santiago, Chile; 3 Department of Population Health, New York University School of Medicine, New York, NY USA; 4 Center for Gun Policy and Research, Johns Hopkins Bloomberg School of Public Health, Baltimore, MD USA; 5 Violence Prevention Research Program, Department of Emergency Medicine, UC Davis School of Medicine, Sacramento, CA USA

**Keywords:** Policy, Firearms, Violence

## Abstract

**Background:**

Comprehensive background check (CBC) laws extend background check requirements to private party firearm transfers to prevent firearm acquisitions by prohibited persons. The aim of our study was to estimate the association between CBC policies and changes in background check rates for firearm acquisition in two states (Oregon and Washington) that have newly-enacted CBC policies.

**Methods:**

We used data on handgun background checks from January 1999 to December 2018 from the National Instant Criminal Background Check System. Observed trends in exposed states were contrasted with counterfactual trends estimated with the synthetic control group method.

**Findings:**

CBC policies were associated with increases in background checks in Oregon (by 18.0%; *p* = 0.074), but not in Washington (4%; *p* = 0.321). A gradual increase in private party checks was seen following enactment in Washington; however, firearm transactions coded as “private” represent less than 5% of total background checks in that state.

**Conclusions:**

Comprehensive background check policies appear to be effective in increasing pre-firearm-sale background checks in Oregon but not in Washington. Differences appear to be related to variations in the proportion of firearm sales that are private party transfers and to gradual adaptation to the new law by private gun sellers.

## Introduction

Public mass shootings and rising rates of firearm homicide and suicide have given new urgency to efforts to prevent firearm violence in the United States. Comprehensive background check (CBC) policies are one such effort, and they enjoy broad public support (Barry et al. 2018). CBC laws extend background check requirements to private party firearm transfers to prevent firearm acquisitions by prohibited persons. In states without CBC policies, approximately 57% of private party transfers precede without background checks; this decreases to 26% in states where CBC policies are in effect (Miller et al. 2017). Nearly all prohibited persons (95%) who acquire firearms for criminal purposes rely on sellers not required to conduct checks (Vittes et al. 2013).

Studies of CBC laws adopted in the 1990s that did not require a permit or license to purchase firearms have not produced evidence that the laws reduce homicides or suicides (Kagawa et al. 2018; Castillo-Carniglia et al. 2019). Recently-adopted CBC laws did not even reliably increase the overall number of background checks (Castillo-Carniglia et al. 2018). Such an increase would be an expected intermediate outcome if CBC policies are to have their intended effect on firearm violence. In this report, we assess the effect of the enactment of CBC laws on handgun background check rates in two US states, extending our preliminary findings for Washington, which considered 2 post-intervention years through December 2016 (Castillo-Carniglia et al. 2018), and adding data for Oregon.

## Methods

We used monthly counts from the Federal Bureau of Investigation’s National Instant Criminal Background Check System (NICS) from January 1999 through December 2018. Washington implemented its CBC law in December 2014 and Oregon in August 2015. We used the synthetic control group method (Abadie et al. 2010) to assess whether the number of monthly handgun background checks per 100,000 people in each state changed following policy enactment in ways different from those forecasted by state-specific counterfactuals. With this method, synthetic controls for each of the two new CBC states were created from a weighted average of a pool of potential control states—the 28 states that did not implement CBC policies during the study period—that minimizes the root mean squared prediction error (RMSPE) for CBC state outcome measures for the pre-CBC law period. The states with potential of being part of the synthetic controls were: Alabama, Alaska, Arizona, Arkansas, Florida, Georgia, Idaho, Kansas, Kentucky, Louisiana, Maine, Minnesota, Mississippi, Montana, Nevada, New Hampshire, New Mexico, North Dakota, Ohio, Oklahoma, South Carolina, South Dakota, Texas, Utah, Virginia, West Virginia, Wisconsin, Wyoming.

Weights for control states sum to 1 and are estimated based on state-specific values for variables shown to predict background check rates in the two study states during the time before their CBC laws took effect. We again used the following variables (Castillo-Carniglia et al. 2018): percentages of the population between ages 18–24 years, male, Latino, black, having a high school diploma or higher, under the poverty line, or living in urban areas, and median household income, Gini coefficient measure of income inequality, violent crime rate, prevalence of firearm ownership (estimated as the proportion of firearm suicide among all suicides) and values of the outcome at 3 pre-intervention time points: January 1999, 2007, and 2012.

The synthetic control group method does not produce a traditional measure of uncertainty or a test of statistical significance. Inference is based on permutation tests (also called placebo interventions), in which the same analysis is replicated, but the intervention assignment is “permuted” across control states. There is no theoretical reason to expect an effect of a non-existent intervention, but random variation in the post-intervention period can exist. The original results are therefore compared with those from the permutation tests. *P*-values are estimated as the quotient of the number of control states with standardized differences (estimated as post-intervention RMSPE/pre-intervention RMSPE) larger than the treated states and the total number of states in the donor pool.

## Results

States with and without CBC policies exhibited an upward trend in monthly handgun background check rates from early 2000s until 2016–2017 and a small decrease more recently (Additional file 1: Figure S1). Rates were lower in states with CBC policies.

Table 1 displays weights for control states and RMSPEs from synthetic control group comparisons. For Oregon and Washington, eight and five control states, respectively, had nonzero weights. The largest weights for Oregon were assigned to Arizona, New Hampshire, North Dakota, and Montana; for Washington, the largest weights were assigned to Florida, Minnesota, and South Dakota.

**Table 1:** Weights and goodness of fit from the synthetic control analyses for Oregon and Washington

State	Oregon	Washington
Alabama	0	0
Alaska	0	0.051
Arizona	0.205	0
Arkansas	0	0
Florida	0	0.167
Georgia	0	0
Idaho	0	0
Kansas	0	0
Kentucky	0	0
Louisiana	0	0
Maine	0	0
Minnesota	0	0.448
Mississippi	0	0
Montana	0.122	0
Nevada	0	0
New Hampshir	0.178	0
New Mexico	0.149	0
North Dakota	0	0
Ohio	0.071	0
Oklahoma	0.078	0
South Carolina	0	0
South Dakota	0	0.307
Texas	0	0
Utah	0	0.027
Virginia	0	0
West Virginia	0.100	0
Wisconsin	0	0
Wyoming	0.096	0
RMSPE	11.3	11.6

*RMSPE* root mean square prediction error

**Fig. 1:**
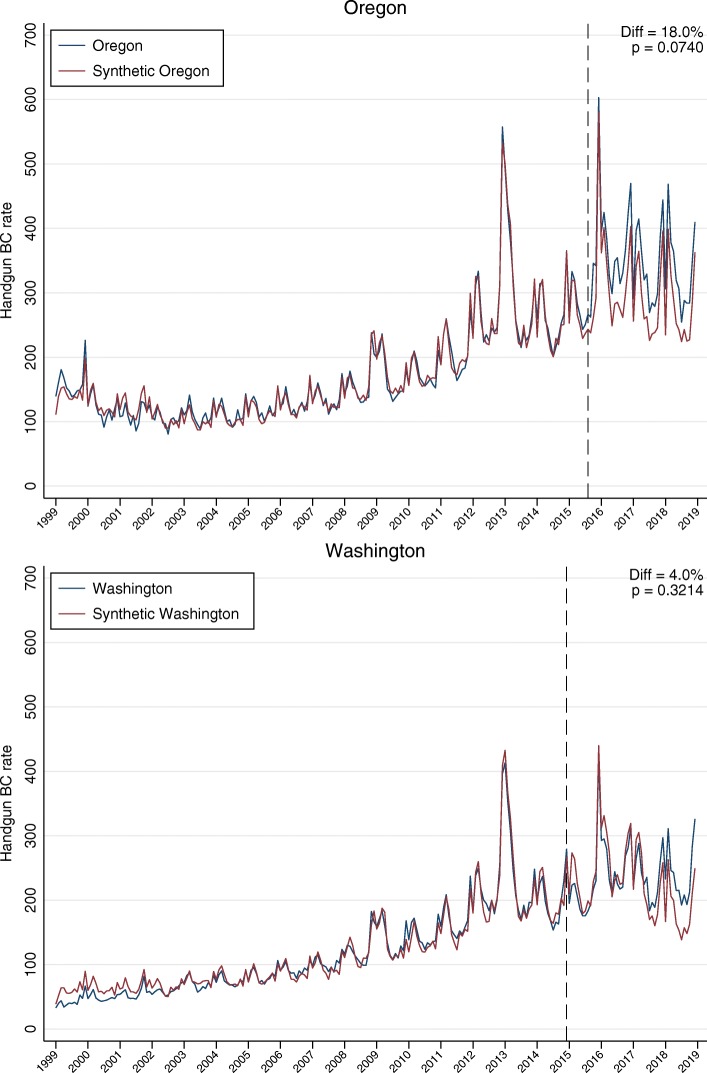
Trend in monthly handgun background check rates per 100,000 people in Oregon and Washington and their synthetic controls, January 1999 to December 2018. Note. *P*-value computed as the proportion of states in the donor pool with standardized change (post-intervention RMSPE/pre-intervention RMSPE) ≥ the standardized change in the treated state. Abbreviation: Diff = Percentage difference

**Fig. 2:**
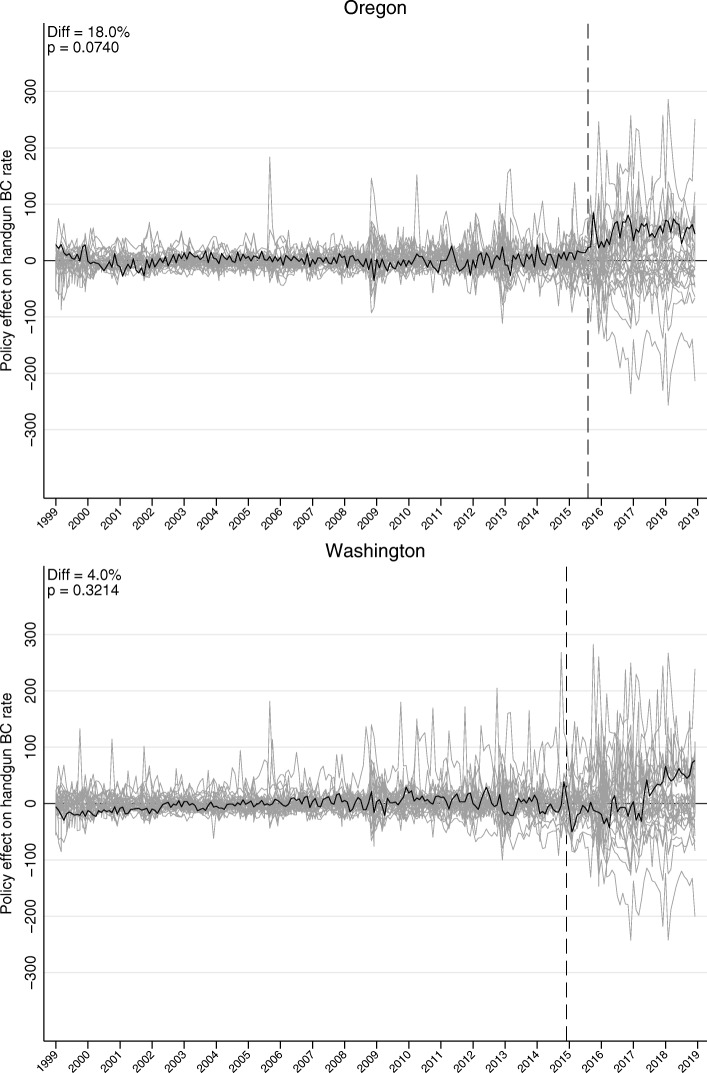
Effect of comprehensive background check policies on monthly handgun background check rate per 100,000 people in Oregon and Washington, January 1999 to December 2018. Note: Effect estimated as the difference between the treated states and their synthetic controls (black lines). Grey lines present the placebo effect for each of the control states in the donor pool

There was a good match between the pre-intervention trend in background checks in each of the new CBC states and its synthetic control (Fig. 1). Further detail is in Table 2 and Fig. 2. The observed average background check rates in the post-intervention period, and the counterfactual rates for the two case states are presented in the first and second rows of the table. The estimated absolute effect of the CBC laws is the difference between the observed and the counterfactual rates, presented in the third row. The last row shows the number of control states with a difference associated with a hypothetical CBC policy greater than the observed difference in the case state. CBC law enactment was associated with a background check rate increase of 52.8 per 100,000 population in Oregon (an 18.0% increase); only 2 out of 27 states in the comparison pool experienced an increase greater than Oregon’s. No change was observed for Washington (4.9%; *P* = 0.3214).

**Table 2:** Change in handgun background check rate (per 100,000 People) after implementation of CBC policies in Oregon and Washington, January 1999 to December 2018

Measure	Oregon^a^	Washington
Observed mean rate of BCs in treated states following implementation	349.9	237.7
Counterfactual mean rate of BCs following implementation	297.1	228.6
Absolute difference in BC rate following implementation^b^	52.8	9.1
Estimated % difference in BC rate following implementation^c^	18.0	4.0
N states in the donor pool with change ≥ treated state (*n* = 28)^d^	2	9

*CBC* comprehensive background checks, *BCs* background checks

^a^ Permutation test for Alaska did not converged, resulting in 27 control states. ^b^ Mean difference in background check rate between the treated states and their synthetic control after policy implementation. ^c^ Percentage difference in background checks rate in reference to BC rate of the synthetic controls after policy implementation. ^d^ Results from permutation test, measured as the post-intervention RMSPE/pre-intervention RMSPE

Washington did experience an increase in checks arising from private-party sales following enactment of its CBC law (Additional file 1: Figure S2). However, checks for private-party transfers accounted for only 3.4% of background checks during the study period; 1.3% in the first year following implementation and 4.8% in the last year available.

## Discussion

Inconsistent association between CBC laws and the overall rate of background checks in these two neighboring states may reflect differences in the proportion of firearm transfers that occur between private parties, in compliance (i.e., adaptation to the new law by private gun sellers), and implementation (e.g., mechanism to detect prohibited persons through integrated data systems, police enforcement) (Vittes et al. 2013; Crifasi et al. 2018). For example, while Oregon showed a consistent increase in handgun background checks following CBC implementation, Washington only experienced a modest nonsignificant increase in background checks by mid-2017, almost two years after implementation of the law. Further comparisons between these two states on factors potentially associated with the effects of CBC policies on background checks such as measures of enforcement (e.g., persons charged with CBC law violations) would be very helpful.

The new laws may have had effects that we were unable to measure or detect. For example, Washington’s increase in private-party checks after its CBC policy took effect suggests a gradual adaptation to the new law by private gun sellers, which warrants the use of longer post-intervention data when evaluating CBC policies. In the case of Washington, however, these checks accounted for less than 5% of total handgun background checks, which is still a very small proportion compared with what has been estimated at the national level (Miller et al. 2017). No pre-enactment data were available, and the increase cannot be attributed to the policy with any certainty.

In contrast to the CBC laws studied here, permit-to-purchase (PTP) laws require handgun purchasers to apply for purchase permits directly to law enforcement agencies who perform background checks and ensure that applicants meet all eligibility requirements (e.g., safety training). PTP laws have been associated with reduced firearm homicides and suicides (Rudolph et al. 2015; Hasegawa et al. 2019). Further research will be needed to determine whether newly-enacted CBC laws in our study states and Vermont (enactment in April 2018) (Vermont General Assembly 2018) are associated with similar benefits.

CBC laws have inconsistent effects on background checks, potentially by several mechanisms acting jointly. All these mechanisms, along with incomplete reporting of prohibiting events, may be responsible for the observed modest or absent effect of CBC laws on rates of firearm violence (Wintemute 2019). Identifying and implementing measures to improve their effectiveness is an urgent public health priority.

## Supplementary information

**Additional file 1:** **Figure S1.** Trend in the rate (per 100,000 people) of monthly handgun background checks in the United State by CBC status, 1999 – 2018. **Figure S2.** Trend in the rate (per 100,000 people) of monthly private party handgun background checks in Washington, July 2013 to December 2018.

**Additional file 2.** Stata code for synthetic control group method and figures.

## Data Availability

Software code used in Additional file 2. The datasets analyzed during the current study are available in the Federal Bureau of Investigation’s National Instant Criminal Background Check System at https://www.fbi.gov/file-repository/nics_firearm_checks_-_month_year_by_state_type.pdf/view
