# Supplementary material for: Effect on background checks of newly-enacted comprehensive background check policies in Oregon and Washington: a synthetic control approach
Source: Inj Epidemiol. 2019 Nov 27;6:45. doi: 10.1186/s40621-019-0225-8 (PMC6880454; doi:10.1186/s40621-019-0225-8)
Supplement: Supplementary file 1 — Additional file 1: Figure S1. Trend in the rate (per 100,000 people) of monthly handgun background checks in the United State by CBC status, 1999 – 2018. Figure S2. Trend in the rate (per 100,000 people) of monthly private party handgun background checks in Washington, July 2013 to December 2018. [file 40621_2019_225_MOESM1_ESM.docx]

**Additional file**

**Effect on background checks of newly-enacted comprehensive background check policies in Oregon and Washington: a synthetic control approach**

Alvaro Castillo-Carniglia^1,2,3^, Daniel W. Webster^4^, Garen J. Wintemute^5^

^1^ Society and Health Research Center, Universidad Mayor, Santiago, Chile.

^2^ School of Public Health, Universidad Mayor, Santiago, Chile.

^3^ Department of Population Health, New York University School of Medicine. New York, NY, United States.

^4^Center for Gun Policy and Research, Johns Hopkins Bloomberg School of Public Health, Baltimore, MD, United States.

^5^Violence Prevention Research Program, Department of Emergency Medicine, UC Davis School of Medicine, Sacramento, CA, United States.

*Correspondence to:*

Alvaro Castillo-Carniglia PhD, MSc.

Society and Health Research Center, Universidad Mayor, Chile.

Badajoz 130, office 1306, Las Condes, Santiago, Chile.

Phone (+56 2) 2518 989826

Email: [alvacasti@gmail.com](mailto:alvacasti@gmail.com)

**Figure S1. Trend in the rate (per 100,000 people) of monthly handgun background checks in the United State by CBC status, 1999 - 2018**

Abbreviations: CBC = Comprehensive background checks. BCs = Background checks

**Figure S2. Trend in the rate (per 100,000 people) of monthly private party handgun background checks in Washington, July 2013 to December 2018**
